# Ultrasound biomicroscopy of hyperpressurized eyes following pars plana vitrectomy

**DOI:** 10.3892/etm.2013.1206

**Published:** 2013-07-05

**Authors:** NA WU, HONG ZHANG

**Affiliations:** 1 Department of Ophthalmology, Tianjin First Center Hospital, Tianjin 300192, P.R. China; 2 Department of Ophthalmology, Tianjin Medical University Eye Hospital, Tianjin 300384, P.R. China

**Keywords:** intraocular pressure elevation, vitrectomy, postoperative

## Abstract

Early elevated intraocular pressure (IOP) following pars plana vitrectomy is a common complication of vitreoretinal surgery and severe pressure elevation may result in visual loss. To investigate the mechanism of IOP elevation following pars plana vitrectomy, a retrospective review of 119 patients (132 eyes) who had undergone vitreoretinal surgery was performed. Ultrasound biomicroscopy (UBM) was used to observe the changes in the structure of the anterior segment following vitrectomy and to compare various parameters pre- and postsurgery. The UBM examination revealed inflammation within the anterior chamber and hyphema with increased IOP. In certain patients, the iris had adhered to the trabecular meshwork and the anterior chamber angle was closed. Cyclodialysis involving the pars plicata and iris was also observed. Furthermore, silicone oil emulsification in the anterior chamber angle and posterior chamber presurgery were noted in certain cases. Edema and forward rotation of the ciliary body resulted in the closure of the anterior chamber angle. The measured parameters indicated that the anterior chamber became shallower and that the anterior chamber angle was narrowed in phakic eyes with elevated IOP. Eyes with elevated IOP and intraocular lenses were not observed to be different from phakic eyes with elevated IOP. This may be due to the fact that an eye with an intraocular lens is thinner than a phakic eye. This study suggests that UBM examination is useful for investigating the pathogenesis of elevated IOP following vitrectomy, and provides a theoretical basis.

## Introduction

Early elevated intraocular pressure (IOP) following pars plana vitrectomy frequently complicates vitreoretinal surgery. Ultrasound biomicroscopy (UBM) is a non-invasive diagnostic procedure, developed in order to achieve superior visualization of the anterior segment of the eye. The aim of this study was to investigate the mechanism of IOP elevation following pars plana vitrectomy. The ultrasound biomicroscope is an imaging instrument used in clinical opthalmology that was developed by Palvin in 1990 for use in clinical ophthalmology research (1). Not only does it enable the anterior segment to be clearly visualized during surgery, it also provides quantitative measurements, which are an important basis for the analysis of certain physiological eye processes and the pathological mechanisms of certain eye diseases. With the advantages of being non-contact, non-invasive and non-interfering, UBM is considered to be a superior imaging detection technique for studies regarding anterior segment morphology (2,3). A previous study concerning the mechanisms and risk factors of high IOP in posterior vitreous resectioning neglected the observation of the anterior chamber angle structure, which is closely associated with glaucoma (4). Comparison of the pre- and postsurgical anterior segment structure angle may help to explain certain mechanisms involved in the increase of IOP, and further provides a theoretical clinical basis.

## Subjects and methods

### Subjects

From January 2009 to January 2011, 119 patients (132 eyes) who underwent a posterior vitrectomy at Tianjin First Center Hospital (Tianjin, China) experienced early postoperative ocular hypertension. With an average age of 46.5 years, there were 66 males (75 eyes) and 53 females (57 eyes) aged between 19 and 72 years. In all the patients who had undergone posterior vitrectomy, the possibility of various primary eye diseases, such as primary or secondary glaucoma, a medical history of ocular hypertension or a family history of glaucoma, were excluded. This study was conducted in accordance with the Declaration of Helsinki, and with the approval of the Ethics Committee of Tianjin First Center Hospital. Written informed consent was obtained from all participants.

### General patient information

The clinical data of the patients were gathered for retrospective analysis, including general patient characteristics. In 132 eyes, there were 43 eyes with diabetic retinopathy (DR) and vitreous hemorrhaging; 24 eyes with DR and tractional retinal detachment (RD); 32 eyes with RD and proliferative vitreoretinopathy (PVR) above grade C2; 10 eyes with RD and PVR less than grade C2; three eyes with traumatic RD; five eyes with traumatic vitreous hemorrhaging; five eyes with branch retinal vein occlusion caused by vitreous hemorrhaging; five eyes with unexplained vitreous hemorrhaging; three eyes with a macular film and two eyes that exhibited vitreous opacity.

### Treatment

All patients were treated with a standard 3-channel closed vitrectomy. According to the condition of the eye, combined phacoemulsification and intraocular lens surgery, stripping surgery, gas-liquid exchange surgery, heavy water injection surgery or laser photocoagulation was selected. Similarly, the type and concentration of padding or filler was selected. The measurement of IOP was conducted using a Schiotz tonometer (Akihito Medical Devices Co., Ltd. Suzhou, China) at the end of the surgery, and the IOP was controlled within the range of 5.5/12.0−7.0 mmHg. All patients accepted a pre- and postsurgical eye examination, including corrected visual acuity, slit lamp, IOP, gonioscopy, ophthalmoscopy and UBM examination. Generally, following surgery, the patients accepted the UBM review within three to seven days, and gonioscopy was required for patients with elevated IOP. We used a P40 Type ultrasound biomicroscope, produced by the Zeiss-Paradigm Company (Hong Kong, China), with a probe frequency of 50 MHz and a scan depth and width of 5 mm. Using the incidental functions of distance and angle measurements (3), we used the UBM instrument in a 12, 3, 6 and 9 o’clock radial scan and saved the captured images of the four quadrants. The main measurement parameters included: i) the depth of the anterior chamber; ii) the thickness of the ciliary body; iii) the angle opening distance 500 (AOD500); iv) the angle between the sclera and the ciliary body; and v) the distance between the trabecular meshwork and the ciliary body.

### Statistical analysis

Statistical data were presented as the mean ± SD. The data were analyzed using SPSS 14.0 software (SPSS, Inc., Chicago, IL, USA) with a t-test to determine whether two groups were statistically different. P<0.05 was considered to indicate a statistically significant difference.

## Results

### Slit lamp and ophthalmoscopy

Of 50 eyes with early postoperative elevated IOP, there were 32 eyes that exhibited eyelid swelling, 26 eyes with corneal edema, 31 eyes with an inflammatory reaction, nine eyes with anterior chamber hemorrhaging and eleven eyes with bloody sediment attached to the corneal wall. In addition, there were four eyes with silicone oil in the anterior chamber, three eyes in which a fibrous membrane had formed at the pupil, two eyes which held an intraocular lens, three eyes that had a barrier of bubbles in the pupil area and two eyes that exhibited partial anterior synechia.

### Gonioscopy

Of the 132 eyes in which the angle of the anterior chamber of the eye was preoperatively observed by angle gonioscopy, 85 had a wide-angle and 47 had an NI angle. Iris morphology was normal in all eyes. No neovascularization was observed in the anterior chamber angle. Postsurgical examination findings within two weeks of surgery were the same those from the preoperative examination.

### UBM

This study compared differences between various measurement parameters pre- and postoperatively in a phakic group and an intraocular lens group (65 and 54 patients, repectively). Due to the low number of cases, an aphakic group was not included in the statistical analysis. In the phakic group, the postoperative anterior chamber depth of the eyes with elevated IOP was significantly different from that in the eyes with a normal IOP (t=2.000, P=0.049). Moreover, the difference between the pre- and postoperative measurements of anterior chamber depth also differed significantly between the elevated and normal IOP groups (t=2.534, P=0.042). In summary, we considered that the postsurgical anterior chamber depth was reduced compared with that preoperatively in the phakic group. Furthermore, the difference in the postoperative AOD500 between the eyes with elevated IOP and those with normal IOP in the phakic group was considered to be statistically significant (t=2.069, P=0.050). Similarly, the difference between the pre- and postoperative measurements of the AOD500 also differed significantly between the elevated and normal IOP groups (t=2.073, P=0.047). The AOD500 is a parameter which reflects the degree of angle width; therefore, the AOD following surgery was markedly reduced in the phakic group with elevated IOP. In our study, in a pre- and postoperative comparison of the distance between the ciliary body and the trabecular meshwork and the angle between the sclera and the ciliary body, no statistically significant differences were identified between the patients with elevated IOP and those with normal IOP (P>0.05).

However, in the intraocular lens group, the postoperative anterior chamber depth of the eyes with elevated IOP was observed to be statistically significantly different from that of the eyes with normal IOP (t=2.066, P=0.050), but the difference between the pre- and postoperative anterior chamber depth measurements was not shown to differ significantly between the elevated and normal IOP groups (t=0.212, P=0.834). This may be explained by the small size of the intraocular lens compared with the phakic lens and the deeper postoperative anterior chamber depth compared with that preoperatively. Using the t-test, other parameters, such as the AOD500, the distance between the ciliary body and the trabecular meshwork and the angle between the sclera and the ciliary body were not considered to be statistically significant (P>0.05). The difference in the postoperative measurements of ciliary body thickness between the eyes with elevated IOP and those with normal IOP was considered to be statistically significant (t=1.926, P=0.037), but the difference between the pre- and postoperative measurements of the thickness of the ciliary body were not considered to differ significantly according to IOP status (t=1.094, P=0.279). Therefore, it is not possible to conclude that the thickness of the ciliary body increases in patients with elevated IOP following surgery.

## Discussion

With advances in technology, pars plana vitrectomy has become an important treatment for ocular posterior segment disease. However, high IOP is a common clinical complication, which may occur at any time following surgery but is most common after one or two weeks. According to previous studies, the mechanism for early postoperative high IOP following the pathogenesis of vitrectomy includes open-angle and angle-closure mechanisms. Open-angle mechanisms include: intraocular expansion by gas injection, inflammatory substances blocking the trabecular meshwork, silicone oil glaucoma and ghost cell glaucoma. Close-angle mechanisms include: pupillary blocking, ciliary body edema and anterior synechia (5).

**Figure 1:**
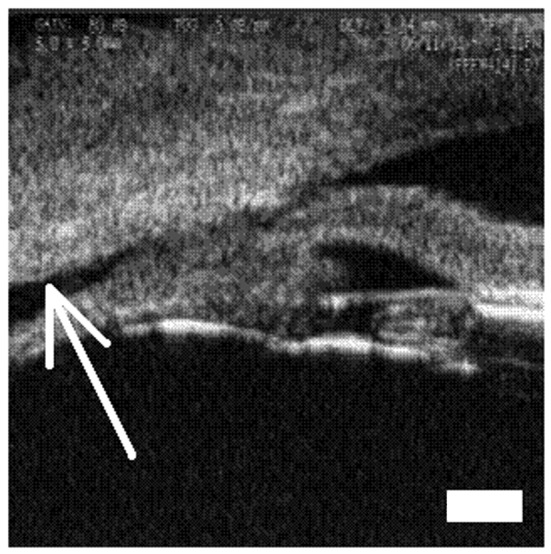
Cyclodialysis involving the pars plicata and iris. The distance is ~1/4–2/3 of the sclera thickness (9 o’clock position).

**Figure 2:**
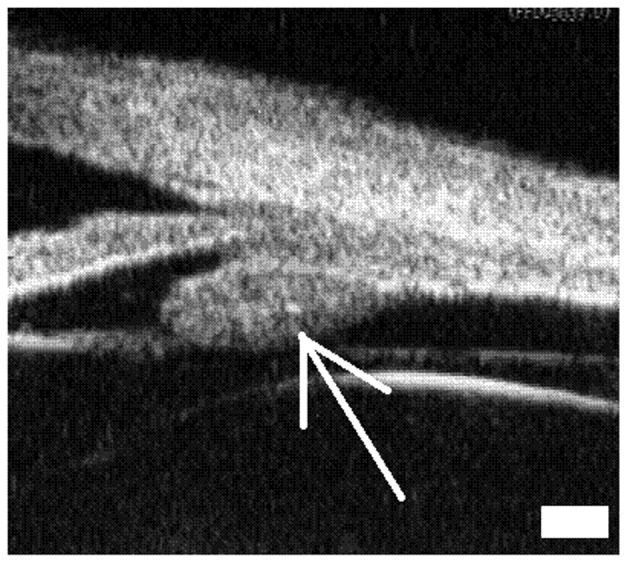
Edema owing to the forward rotation of the ciliary body postoperatively (3 o’clock position).

By the use of UBM, the current study established that there were 16 eyes (12.12%) in the high IOP group in which ciliary body detachment (full 360º) had occurred (Fig. 1). According to a previous study (6), following surgery, a wide-ranging detachment of the ciliary body, edema of the ciliary body, and forward rotation of ciliary processes may create pressure on the peripheral iris, causing it to move to the trabecular meshwork, leading to angle closure, and an increase in the IOP. Furthermore, when the ciliary body is detached, the ciliary body and choroidal liquid push on the ciliary body and the suspensory ligament, which causes the lens to move forward and the anterior chamber to become shallower. In the current study, three eyes in the elevated IOP group exhibited edema of the ciliary body. The swollen ciliary processes had rotated forward to the root of the iris and accumulated in the gap between the iris root and the equitorial lens region, which had moved forward (Fig. 2). Clinically, in certain cases the reduction of IOP was due to surgical trauma where a large area of condensation and the secretion of aqueous humor was affected by the ciliary membrane. Due to a reduction in the IOP, the liquid rapidly accumulated in the suprachoroidal space, which caused the detachment of the choroid. Therefore, choroidal detachment further aggravated hypotony and an inflammatory reaction exacerbated this cycle.

**Figure 3:**
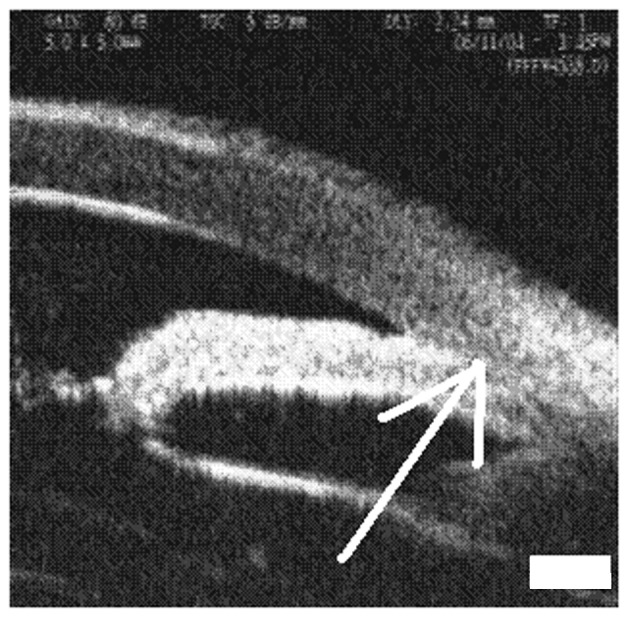
The iris adheres to the trabecular meshwork and the anterior chamber angle (6 o’clock position).

**Figure 4:**
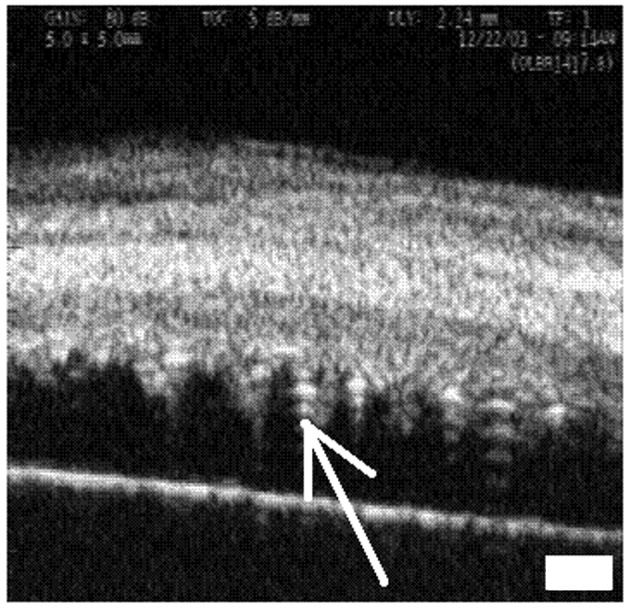
Silicone oil emulsification in ciliary processes (coronal cross section.

Furthermore, the vorticose veins were pressed on during surgery, which blocked the drainage of ciliary body venous blood, resulting in ciliary body edema. This resulted in the swollen ciliary processes reversing forward, so that the peripheral iris pressed against the trabecular meshwork, which aggravated the closed angle and caused an increase in the IOP. Anterior chamber hemorrhaging blocked the peripheral iridotomy opening holes, which in turn blocked the flow of aqueous humor in the anterior and posterior chambers. In addition, aqueous humor accumulated in the posterior chamber, which caused intravitreal silicone oil to protrude into the pupil area, resulting in the formation of a pupil block (Fig. 3), and thereby the occurrence of elevated IOP (7). Azzolini *et al*(8) considered that silicone oil droplets may display a strong reflection ring, and floating and emulsified silicone oil droplets were due to the lack of a reflection ring. Due to their light weight, silicone emulsion droplets were able to float up and move closer to the cornea; therefore, floating, small, highly reflective and clear-cut drops of silicone oil were observed in the anterior chamber. When moving closer to cornea, the silicone oil droplets may be observed as solidified matter in the corneal endothelium (9). In the current study, there were two eyes in the high IOP group with secondary surgery in which emulsified silicone oil droplets were visible on the iris surface under the slit lamp prior to surgery. UBM examination revealed fine oil droplets attached to the corneal endothelium, and indicated that the angle of the anterior chamber had opened (Fig. 4). Therefore, the emulsified silicone oil droplets and phagocytic macrophages blocked the trabecular meshwork, which led to an increase in the IOP (10).

According to the comparison between the eyes with high IOP and those with normal IOP in the phakic group, the anterior chamber depth became shallower and the AOD reduced following surgery; however, it is not possible to conclude that early postoperative elevated IOP has a direct relationship with this condition. In the intraocular lens group, every preoperative and postoperative measurement parameter exhibited no significant change, which may be related to the fact that the thickness of the human lens is 4.0 mm, while the thickness of an intraocular lens is only 0.8 mm. Combined with phacoemulsification and intraocular lens implantation, the anterior chamber depth is likely to increase following surgery.

In conclusion, the mechanism of early postoperative elevated IOP following vitrectomy is complicated. This study identified that the possible mechanism of early postoperative ocular hypertension by UBM comprises the following: postoperative edema of ciliary body; the anterior chamber becomes shallower and is rotated forward; and the increase in IOP is caused by angle narrowing. Every measurement parameter suggested that the AOD decreased and the anterior chamber became shallower in the phakic IOP group following surgery.
